# ENGAGING LOW-INCOME SENIORS IN PARTICIPATORY DESIGN OF SMART SPEAKER APPLICATIONS FOR WELLNESS

**DOI:** 10.1093/geroni/igac059.1001

**Published:** 2022-12-20

**Authors:** Jane Chung, Jodi Winship, Tracey Gendron, Rachel Wood, Natalie Mansion, George Demiris

**Affiliations:** Virginia Commonwealth University, Richmond, Virginia, United States; Virginia Commonwealth University, Richmond, Virginia, United States; Virginia Commonwealth University College of Health Professions, Richmond, Virginia, United States; Bon Secours Mercy Health System, Cincinnati, Ohio, United States; Virginia Commonwealth University College of Health Professions, Richmond, Virginia, United States; University of Pennsylvania, Philadelphia, Pennsylvania, United States

## Abstract

Low-income senior housing (LISH) residents are at a high risk of unmanaged health conditions, loneliness, and limited healthcare access. Smart speakers have the potential to improve wellness in LISH settings. We conducted a user-centered process with primarily African American, LISH residents (N=25) to develop prototypes of smart speaker applications for wellness and social connections. Five focus groups were conducted to elicit feedback about challenges with maintaining wellness and attitudes towards smart speakers. Participants expressed their desires for using the technology for safety and health. Through design workshops, they identified several smart speaker functionalities perceived as necessary for improving wellness and social connectedness. Then, seven low-fidelity prototypes and scenarios were developed in the following categories: wellness check-ins, befriending the virtual agent, community involvement, and mood detection. We demonstrate how smart speakers can provide a tool for their wellness and increase access to applications that provide a virtual space for social engagement.

